# A Case of Metastatic Melanoma in the Ureter

**DOI:** 10.1155/2016/1853015

**Published:** 2016-10-12

**Authors:** James Macneil, Tania Hossack

**Affiliations:** Department of Urology, Westmead Hospital, Westmead, NSW, Australia

## Abstract

Advances in the treatment of melanoma are resulting in patients living for extended periods after being diagnosed with metastatic disease. Metastases to the ureter are rare, but they have been described in the literature on a number of occasions. In this case report, we describe a patient with established metastatic melanoma who, whilst taking and responding to immunomodulatory therapy, was found to have an obstructive mass in the middle of his left ureter. Rather than performing either a nephroureterectomy or partial resection of the ureter, we opted to perform an endoscopic resection of the melanoma. Follow-up imaging at 12 months shows no evidence of local disease recurrence. We submit that primary endoscopic management of metastatic melanoma in the ureter is a viable alternative to a radical approach.

## 1. Introduction

Melanoma is a common skin cancer with a high incidence in Australia and elsewhere [1]. The prognosis for melanoma varies dramatically based on both the primary histopathology and a number of prognostic factors including gender, location of the primary tumour, and the results of sentinel lymph node biopsy [2]. Although metastatic melanoma was previously uniformly lethal, the advent of new therapies including advanced chemotherapeutics and immunomodulatory drugs is substantially improving the prognosis [3].

Metastatic spread of melanomas to the genitourinary system is a recognised but rare complication [4–7]. The improvement in prognosis of metastatic disease poses the challenge of how to manage the locoregional effects of a metastatic deposit in an individual with an extended life expectancy.

This case report considers a case of a metastatic melanoma deposit in the ureter managed with the intent of preserving renal function whilst maximising quality of life. As a Stage IV melanoma, we accepted that the intent of this resection was noncurative.

## 2. Case Report

Our patient is a 32-year-old otherwise well man who was initially diagnosed with melanoma following excision of a 5 mm naevus from his back in June 2012. The primary pathology showed it to have a Breslow thickness of 5 mm, a Clark level of 4, no evidence of ulceration, and a mitotic count of 7/mm^2^. Subsequent gene testing demonstrated the tumour to be BRAF positive. On the basis of this poor pathology, a Wide Local Excision and sentinel node biopsy were performed. The positive nodal biopsy resulted in the patient having an axillary clearance (5 out of 30 nodes positive). He had adjuvant radiotherapy (48 Gy to the axilla) and he was enrolled in a clinical trial which involved the administration of two immunomodulatory drugs: Nivolumab and Ipilimumab.

On a routine staging CT-abdomen/pelvis in December 2014, the patient was first noted to have a mild left-sided hydroureter. Neither the CT scan nor ultrasound performed soon after demonstrated a clear cause. A repeat CT-abdomen/pelvis in March 2015 showed the development of a 6 mm nodule in the mid left ureter at approximately the level of L4 vertebrae. A PET Scan performed at the time did not suggest it to be a metastatic deposit. A further repeat CT was performed in May 2015 by which time the nodule had progressed to being 10 × 9 mm and the hydroureter had progressed significantly. Throughout this period, serial measurements of renal function did not demonstrate any abnormalities, with a stable Creatinine between 90 and 100 *μ*mol/L. The patient denied haematuria, and urinary cytology was negative. At this time, another metastatic deposit was found (a metastatic deposit in the left parafalcine region of the brain, subsequently treated with stereotactic radiotherapy) in addition to apparent lymphadenopathy.

On the basis of evidence of the progression of the nodule and increasing hydroureter, the patient was referred to the Uro-Oncology Clinic at the Crown Princess Mary Cancer Centre, Westmead. In light of the position of the nodule in the mid ureter, the decision was made to attempt an endoscopic resection.

**Figure 1:**
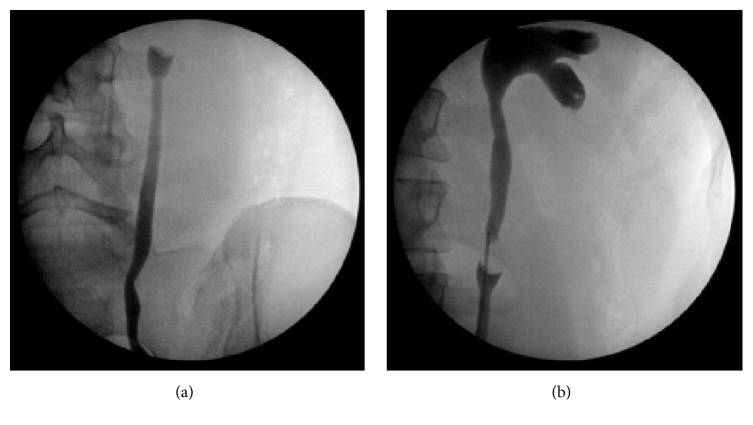
Images from the retrograde pyelogram performed during the initial ureteroscopy and resection. Note that no contrast is passing above the mass (a) and the filling defect from the mass (b) once the ureteric catheter is passed around it.

The patient underwent ureteroscopy at Blacktown Hospital in July 2015. The retrograde pyelogram confirmed the presence of the lesion, with evidence of obstruction in the mid ureter above the lesion (Figure 1). Direct visualisation demonstrated a large ovoid mass attached by a thin stalk to the ureteric wall. This stalk was lasered, and the mass retrieved in three pieces by a basket through a sheath in order to minimise the risk of seeding into the lower tract. Since the depth of the lesion was unknown and the stork had a narrow base, the lesion was lasered full thickness in order to ensure maximal clearance. Low pressure irrigation was used throughout. Macroscopic inspection did not suggest involved margins, and completion ureteroscopy and pyeloscopy did not demonstrate any further suspicious lesions. In order to avoid the risk of causing a postoperative stricture, a ureteric stent was placed at the end of the case.

Histopathology of the retrieved fragments confirmed the diagnosis of the mass being a metastatic melanoma.

**Figure 2:**
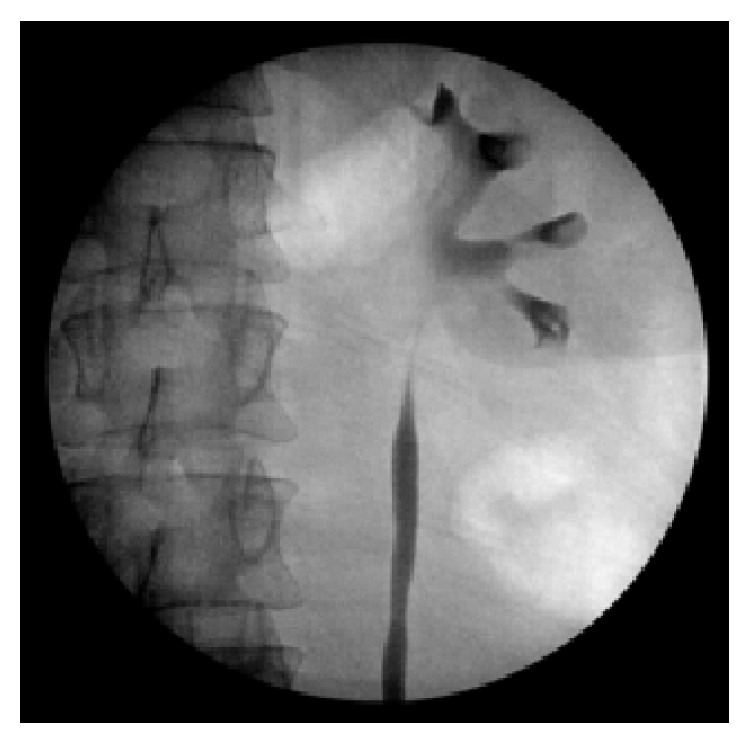
Retrograde pyelogram conducted at the time of the relook ureterorenoscopy. Note the absence of filling defects and the normal calibre upper tracts.

Following removal of the stent, further ureteroscopy was performed in October 2015. The retrograde pyelogram did not demonstrate any filling defects or any evidence of the hydroureter (Figure 2). Direct inspection did not demonstrate any evidence of the metastatic deposit or its stalk or any further metastatic deposits.

At patient's most recent follow-up in late July 2016, there is no evidence of local disease recurrence. His serial staging CT-abdomen/pelvis remains clear of any hydroureter, the apparent lymphadenopathy has resolved, and his renal function remains stable (Creatinine 100 *μ*mol/L). He remains clinically well.

## 3. Discussion 

Metastatic melanoma deposits in the upper urinary tract have been described on a number of occasions; however, the outcomes in these cases have been poor. In recent case reports, patients have either progressed to nephroureterectomies [6, 7] or have died soon after diagnosis [7]. We therefore did not have an evidence-based approach to planning this patient's treatment.

Underlying our decision making was the changing prognosis for metastatic melanoma. Until the development of immunomodulatory therapies, metastatic melanoma had an extremely poor prognosis with extremely limited life expectancy following diagnosis [2]. However, with the development and use of these drugs, there is reason to be cautiously optimistic regarding the medium and longer term prognosis of otherwise well individuals with metastatic melanoma. As such, treatment of locoregional metastases should be planned with a view to both the oncological and functional outcomes [8].

Our patient was a prime example of such a patient: despite extremely poor prognostic markers on initial diagnosis, the patient was responding well to treatment and was healthy at the time of the mass in his ureter being found.

On performing the ureteroscopy and retrograde pyelogram and confirming the morphology of the lesion, three potential approaches were available to us: resection of the ureter, an endoscopic resection of the mass, or an ablative approach.

Resection of the ureter has been described in Gakis et al. [6]: partial ureterectomy with an end-to-end anastomosis and wide margins. This approach has advantages from an oncological perspective; however, the location of our patient's nodule (approximately the level of L4 vertebrae on the preoperative imaging) posed a higher risk of complications (such as anastomotic leaks and scarring or stricture of the ureter) than the other available approaches. Both the patient and the medical oncology team were cautious regarding a major operation (a partial ureterectomy or nephroureterectomy) owing to the possible impact on patient's quality of life, especially, if there was a moderate possibility of complications necessitating ether extended admissions or further procedures. We also note that there is only modest evidence that metastasectomies provide improvements in either disease free or overall survival [9].

An ablative approach has also been described as in Nair et al. [7] using a laser for multiple lesions in the upper urinary tract. This approach appears to have been used with the intention of managing symptomatic haematuria rather than achieving oncological clearance of the lesions. We elected not to use this approach in our patient for a number of reasons, principally the risk of injury to the ureter, the risk of seeding during the ablation, and difficulty in achieving an adequate clearance whilst lasering a large lesion.

The decision making process encompassed a number of concurrent considerations, including oncological outcomes, functional outcomes, and impact on quality of life in addition to patient preference.

From an oncological perspective, it was accepted that patient's disease had already metastasised to multiple sites and, thus, although each was individually treatable, therapy with curative intent was not appropriate. The patient was however clinically well enough to warrant treatment especially in light of potential compromising his renal function from progressive obstruction from the metastasis (important in considering further treatment).

Therefore, the decision was made to attempt an endoscopic resection. Although, from a purely oncological perspective, this was a less favourable option than a partial ureterectomy or nephroureterectomy, its functional outcomes were clearly superior and it was a far less morbid procedure. It also did not preclude the possibility of performing a nephroureterectomy in the event that there was evidence of locoregional recurrence after the initial resection. We see clear parallels with the accepted standards for managing melanomas in anatomically sensitive areas such as the face where smaller than ideal margins are accepted when performing surgical resection in order to retain function. We note that this is also in line with international guidelines regarding the surgical approach to managing metastatic melanoma [8].

Consideration was given to the risk of seeding in an endoscopic approach. The approach adopted aimed to mitigate the risk as far as possible: a sheath was advanced to the lesion, the lesion was retrieved in the smallest number of pieces possible through the sheath, and low pressure irrigation was used throughout. Given the presence of metastatic disease elsewhere, we felt justified in accepting somewhat more risk in this regard compared to what we might have done in the absence of this evidence.

A similar consideration was given with regard to lasering the lesion full thickness. Although lasering the stalk full thickness posed a risk of making a hole into the retroperitoneum, and an associated risk of seeding, it would also result in a substantial improvement in the chances of local clearance of the metastatic deposit. And, with evidence of metastatic disease elsewhere, we felt that the risk of retroperitoneal spread was less clinically significant than the risk of local recurrence and functional impairment.

Our follow-up so far validates our approach: functionally, the patient has done very well (the hydroureter has resolved well, and his renal function has remained stable throughout) and, from an oncological perspective, repeat ureterorenoscopy has not found any macroscopic evidence of disease recurrence.

Our plan is to monitor the patient through repeat imaging and serial renal function testing and to respond to any evidence of locoregional recurrence as it occurs.

## 4. Conclusion

Although it is difficult to generalise, particularly in such a rare condition, we believe that the approach used with this patient is a valid approach to consider in similar presentations. Given the improving medium and longer term survival of metastatic melanoma patients, it would be reasonable to expect that further cases of melanoma metastasising to the upper urinary tract will be found. As such, we would recommend performing primary endoscopic evaluation of the metastases, with consideration given to endoscopic resection and close monitoring in appropriate patients.
